# Exploring the Impact of Home-Based Support on Informal Caregivers: A Qualitative Study in Hyderabad, Pakistan

**DOI:** 10.7759/cureus.100733

**Published:** 2026-01-04

**Authors:** Sughra Mahnoor Mangrio, Syeda Areesha Ali Naqvi

**Affiliations:** 1 Health Sciences, Health Services Academy, Islamabad, PAK; 2 Health Systems and Policy, Health Services Academy, Islamabad, PAK

**Keywords:** emotional burden, gender roles, home-based care, informal caregiving, pakistan

## Abstract

Background

An aging population in Pakistan is relying more and more on informal caregivers as organized elder care systems are hard to come by. In cities such as Hyderabad, caregiving is largely a family-oriented responsibility, and it is deeply rooted in culture and religion. Women disproportionately shoulder the burden of caregiving and experience emotional, physical, and financial distress as a result. While the global literature acknowledges caregiving as fundamental to health systems, there is scant research evidence on the experiences of caregivers in low-resource South Asian settings. This paper explores the difficulties, coping strategies, and social relations shaping informal caregiving in Hyderabad, Pakistan.

Methods

A qualitative explorative research design was followed. The data were collected by using semi-structured in-depth interviews from 11 family caregivers who render home-based unpaid care to an older adult or chronically ill family member. Patients were selected conveniently from the Civil Hospital, Hyderabad. The interviews were carried out in Sindhi and Urdu, transcribed verbatim into English translation, and subjected to thematic analysis. The research was ethically approved, and informed consent was obtained from the Higher Education Commission (HEC) Institutional Review Board.

Results

Six major themes emerged from the analysis: caregiving roles, family involvement, decision-making in care, health system experiences, financial difficulties, and psychosocial effects. The caregivers, particularly women, have emotional burnout, fatigue, social isolation, and financial strain: the results say. Spouses, sons, and brothers (many of whom comprise the pool of male relatives) were more likely to have provided economic aid than volunteer assistance. Along the same line, people complained about access to care: wait times and a bad rapport with doctors. However, caregivers described moral satisfaction and a kind of emotional connection as core factors keeping them going.

Conclusion

Unpaid caregiving in Pakistan is a kaleidoscope of cultural coercion, gender inequality, and institutional betrayal. In addition, policy responses such as caregiver training, respite care, financial assistance, and mental health services, which have been offered to soldiers who return from war zones, have been created to alleviate the strain of caregiving and provide better outcomes for eldercare. This research provides policy-relevant information for healthcare decision-makers and contributes to the global geriatric care discussion.

## Introduction

Caregiver contribution in the health sector is undervalued and yet crucially instrumental, especially in low-middle-income countries (LMICs) such as Pakistan, where informal care is a significant component for individuals living with chronic illness, functional impairment, or old age. Care recipients are then dependent on unpaid informal carers, typically family members, to provide care for their daily health condition and emotional well-being [1]. Their contributions are invaluable, especially in nationally underfunded and overextended public health systems. However, caregiving is a burden on the physical, emotional, and financial health of whoever provides it, and the consequence when that burden goes unaddressed can be burnout, declining mental health, and less capacity for more labor. Understanding these nuanced needs for one's role is important to support caregiver well-being and patient outcomes in family-centered care systems [2].

Care, in Pakistan, is a social liability grounded on religious values and traditional cultural norms, which prioritizes compassion, filial responsibility, and collective senior familial-based responsibilities within the family system. Such norms present caregiving as morally/spiritually required and frequently elevate the former to a self-gift rather than as a social duty [3]. Thus, the care burden is disproportionately on women who have to subsume their educational and work pursuits under that of nurturing. This sexed division of caring work reinforces economic and emotional imbalances, as many female informal carers receive low social recognition and institutional resources. No acknowledgment that caregiving is skilled labor (not even a family-friendly perk) further contributes to the invisibility of caregivers in healthcare policy and programming, which explains the lack of system support [4].

In a country such as Pakistan, care is further complicated by the stigmatization of formal care. The use of external help is generally seen as a form of neglect or failure on the part of the family, and they are reluctant to access institutional support. This trend has led to a growing dependence on private, unpaid informal home care that operates beyond professional health service provision [5]. Hyderabad is just one place where the caregiving fabric is transforming; in cities, old forms of care are being strained by migration, mounting health expenses, and changing social mores. Despite loving care early on the journey, so much of this is stripped away as joint family systems break down and another person takes on more and more by way of complex medical and emotional needs.

Given that informal caregiving is such an important yet often hidden aspect of the global healthcare system, in its clear communication, caregivers are the backbone of long-term care, and this is particularly true in low-middle-income countries (LMICs) where formal health systems are weak. Their volunteers are not only helping to keep people well but also relieving pressure on overburdened health systems around the world. Tending to the chronically ill, sick, and elderly without significant institutional backing is common practice among home caregivers in Pakistan [6]. Despite the vital task of caregiving, most caregivers have a high level of stress, anxiety, and depression as a result of the long time providing care [7-9]. This psychological toll can be compounded by the physical and financial hard work of caretaking, especially in individuals who need to quit a job or educational training to care full time at home.

So far, global caregiving evidence has largely been generated from Western contexts where formal care systems and social protection mechanisms are more established. This focus leaves a major gap in the understanding of caregiving within South Asian communities, wherein care is experienced as nested within complex social and cultural gendered frames. A few small studies, conducted mostly in Pakistan and the South Asian region, only identified that caregivers face structural and social barriers such as the lack of institutional respite services, financial uncertainties, and not getting value for their emotional labor [10]. Furthermore, according to prior studies, it is women (not men) who consider caregiving as a societal duty and who see both the social obligation and personal price of giving care [11]. These findings underscore the essential need for regionally tailored research that considers caregiving in its cultural and economic context.

In this regard, the sixth biggest city of Pakistan, Hyderabad, has become a pertinent objective for exploration. Like many other densely urbanized cities, the rest of traditional caregiving messages contrast with the emergence of new health problems associated with modernization and rural-urban migration. Caregiver families are frequently straddling maintaining cultural norms around care and fitting in with contemporary expectations, which constrains time for care and resources [12]. The great diversity in the city, massive health disparities, and changing family structure make it an ideal setting for seeing how challenges of caregiving are shifting in Pakistan.

The available literature on the topic is scanty, and this study intends to contribute to filling this void by investigating the experiences of informal caregivers in Hyderabad, Pakistan. It explores the physical, emotional, and financial costs of caregiving borne by caregivers, as well as how cultural, gendered, and societal norms impact their practices of care [13]. This is supplemented by an exploration of the coping and resiliency strategies of carers, highlighting the interplay between cultural norms and psychological well-being. The study also adds to the international dialogue on caregiving by focusing attention on sociocultural aspects of care in low-resource settings. The results, in short, seek to guide culturally relevant policies and interventions that improve home-based caregivers' support systems and caregivers' well-being in Pakistan.

## Materials and methods

Study design

This is a qualitative study to investigate the experiences of informal home-based caregivers receiving care and medication support from family members in Hyderabad, Pakistan. An exploratory and descriptive design was applied to describe the emotional, physical, and social dimensions of caregiving in a cultural/religious context [14]. This design was appropriate to investigate contextualized, nuanced, and subjective experiences of caregiving against a "background" where little is known about those phenomena. In-depth, semi-structured interviews were conducted to provide thick and contextualized descriptions. This method allowed the participants to provide their viewpoints freely and led the conversation using open-ended questions. The interviews were carried out in the participants' native languages (Sindhi and Urdu) to facilitate and also ensure that the participants felt at ease, as well as to maintain the credibility of the information shared. Interviews were all audio-recorded, fully transcribed, and translated into English to ensure the participants' voices and the meaning within them remained during the analysis process.

Study setting

The study was carried out at the Civil Hospital, Hyderabad (Liaquat University Hospital {LUH}) [15]. LUH is a public sector tertiary care hospital situated in Central Hyderabad, Sindh. It was established in 1881 with a combined capacity of around 2,000 beds at its Hyderabad and Jamshoro campuses. The branch in Hyderabad, where this research project was conducted, caters to urban and semi-urban populations and is conducive to the engagement of caregivers from various socio-economic and cultural contexts. The busyness and approachability of the hospital setting were conducive to exploring caregiving in an impoverished, family-reliant environment.

Study duration

The study was conducted during a six-month period (recruitment, data generation, transcription and translation, thematic analysis, and write-up). Although this duration is relatively short, it provided depth and quality throughout each phase of the research.

Study population and sampling

Informal carers were described as individuals who offer unpaid continuous care, commonly to family members with a long-term disabling and/or age‐related illness. The respondent group was composed of the visiting attendants from the Civil Hospital, Hyderabad, who were managing patients during the study period. Cohort participants were immediate family members (children, spouses, and siblings) who provided care in the melee of things: physical assistance, emotional support, and overseeing medication and care both at home and in the hospital [16]. The participants were recruited through purposive sampling of individuals who were eligible for and best situated to illuminate the study aims. Care givers were referred by doctors at the Civil Hospital, Hyderabad.

Eleven caregivers varying in their caregiving relationship and in sociodemographic characteristics were included in the final sample. Thematic saturation was reached at this figure, beyond which no new themes or insights emerged during the remaining interviews. Saturation is a common methodological yardstick in qualitative research, and it usually occurs between the 10th and 15th interview with a relatively homogeneous sample. In the analysis process, through recursive coding, we extracted the depth and range of nuances from the 11 interviews, and the representation was sufficiently rich to support valid thematic analysis.

Given that this was a qualitative exploratory design, a classical statistical power analysis could not be applied. Quantitative hypothesis-testing studies use power analyses to determine the minimum size of a sample required for statistical generalizability. However, in qualitative research, analytic rigor does not depend on sample size; instead, it depends on data saturation, triangulation, inter-coder agreement, and iterative theme validation without any calculation of power in the statistical sense. The method presented in this paper is consistent with previous proposals for sample sizes to be guided by the pursuit of understanding rather than size. Comparable sample sizes have been used in previous qualitative studies related to informal caregiving in Pakistan and across South Asia, so the present sample may be regarded as adequate [3,6,10].

To put the sample in context, national estimates are that 20%-25% of urban Pakistani households have one or more informal caregivers taking care of an older relative who is sick or disabled [3]. This rate of occurrence bolsters the external validity of recruiting subjects from a big urban hospital serving ethnically, socially, and economically heterogeneous caregiving families, such as Civil Hospital, Hyderabad, where families from diverse strata are served free or subsidized care.

Eligibility criteria

Primary caregivers of a family member with a chronic relapsing health condition (CRHC) requiring assistance who were involved in caring for the patient during the study period were included. Exclusion criteria were individuals who did not directly provide care or who could not or would not give informed consent. These criteria resulted in a sample of participants who had sufficient caregiving experience for research purposes.

Data collection

Data were collected through semi-structured in-depth interviews inside the hospital's wards or, where possible, homes in Sindhi or Urdu language. Questions in the interviews addressed daily caregiving activities, managing medications, emotional and physical strain, burden on lifestyle due to memory disorder, socio-economic impacts, and coping strategies [17]. All interviews were audio-recorded with the participants' permission, transcribed, and translated into English for analysis. Besides interviews, nonparticipant observations were carried out in order to collect communication events and real caregiving activities, the dancers of care between caregivers and patients, and environmental issues within the hospital. This note provided valuable contextual information that supplemented the interview data.

Data analysis

The qualitative data were analyzed using thematic analysis in an iterative, inductive manner. The researchers first became familiar with transcripts and field notes through several readings. Open coding was carried out subsequently to identify common themes and concepts. These codes were then clustered together as themes that represented the essential experiences of caregiving [18]. The topics were further clarified for internal consistency and relevance to the objectives of the study.

Ethical considerations

The Ethical Review Committee of Health Services Academy, Islamabad, approved the study ethically (approval number: 000543/HSA/MSPH-2022). The participants were advised regarding the aim of the study, confidentiality, and the right to drop out at any time without penalty. Consent was done either orally or in writing according to the preference of the participant. Participant identities were anonymized using pseudonyms, and data were securely archived. The participants showing signs of distress in the course of interviews were provided assistance or directed to suitable counselling services.

## Results

Overview

The objective of this qualitative study was to investigate how the management of health and medication shapes the caregiver-patient relationship in informal caregiving to known patients in Hyderabad, Pakistan. The results, however, presented ongoing tension between multiple roles of caregivers and different emotions and system problems they experienced. Major themes and sub-themes emerged from these experiences and were based on the semi-structured interviews with the 11 informants.

Demographic profile of the participants

Data were collected from 11 family caregivers in Hyderabad. Subjects were represented on a range of socio-economic classes and age distribution (20-45 years), as well as cross-sectional socio-economic strata. There were seven women and four men, as shown in Table 1. Women sponsored hands-on care recipients to a significantly greater extent than men, who performed the role of skeptical financial provider. These patterns were consistent with traditional gender role (including socially constructed) norms in the study population.

**Table 1:** Demographic Analysis of Caregivers in Hyderabad

Participant	Age	Gender	Relationship to Care Recipient	Care Recipient's Condition
1	20	Female	Daughter-in-law	Long-standing diabetes
2	25	Female	Daughter	Arthritis
3	30	Male	Husband	Chronic illness
4	35	Female	Granddaughter	Limited mobility
5	40	Male	Son	Heart disease
6	45	Female	Daughter	Uncontrolled hypertension
7	32	Female	Sister	Vision problems
8	38	Female	Wife	Dementia
9	42	Male	Son	Chronic arthritis
10	28	Female	Niece	Multiple health issues
11	45	Male	Son	General health decline

In that it represented various types of caregivers and caregiving situations, the sample allowed us to tap tasks associated with caregiving in all its diversity, and patterns of how task demands were more or less expansive depending on age, sex, and family configurations. The heterogeneity of the participants provided several perspectives in the study on how caregivers felt emotionally and physically overwhelmed and structurally unable to access healthcare.

The gender distribution reveals that most of the caregiving is performed by women (64%) (Table 1), which highlights the gendered division of caregiving within Pakistani society. The younger caregivers (20‐35 years) cared mostly for the parents or in-laws, with the responsibility increasing over time when comparing it to those aged 40‐45 who were caregiving to older parents, symbolizing the increase of care according to age and family cycle. Lower socio-economic status participants reported being more strained from the lack of paid help and the lack of healthcare. These trends imply that caregiving responsibilities and emotional strain are influenced by both gender and socioeconomic status.

Male caregivers, while underrepresented, were involved primarily in financial coordination and hospital communication tasks; female caregivers undertook hands-on physical and emotional care. This provides a parallel for traditional hierarchies of caregiving that are based on cultural assumptions and the construction of the household. Furthermore, caregivers of patients with a chronic illness, such as dementia or arthritis, reported more physical fatigue and emotional exhaustion than those caring for stable chronic illness (hypertension).

Thematic analysis

The thematic analysis of the interviews revealed six key themes, as given in Table 2.

**Table 2:** Themes and Sub-themes

Theme	Sub-theme	Verbatim Response	Reflection
Caregiving responsibilities	Daily care tasks	I look after my 78-year-old mother-in-law, who has diabetes and arthritis. My day revolves around her needs, from meal prep and medication to helping her with her mobility	Caregivers often devote their entire day to addressing the needs of their care recipients, reflecting the intensity of their responsibilities
Caregiving responsibilities	Emotional commitment	I care for my father and feel it is my duty to ensure his well-being	The emotional bond and sense of duty drive caregivers to prioritize the health of their loved ones
Caregiving responsibilities	Monitoring health changes	I keep track of her blood sugar levels daily and report to the doctor	Caregivers often take on roles that require attention to detail and consistency
Caregiving responsibilities	Managing emergency situations	I have had to rush her to the hospital in the middle of the night more times than I can count	Handling emergencies is a recurring and stressful aspect of caregiving
Role of other family members	Limited male involvement	My brothers assist occasionally, but caregiving is seen as my responsibility since I am the daughter	Cultural expectations often limit the involvement of male family members, placing the burden on women
Role of other family members	Financial support	My husband helps financially, but the daily caregiving responsibilities are mine	While financial contributions are valuable, they do not alleviate the physical and emotional demands on primary caregivers
Role of other family members	Emotional support from siblings	My sister calls daily to check on both me and mom; it really helps emotionally	Family members who offer emotional support can significantly lighten the caregiver's burden
Role of other family members	Intergenerational Conflicts	My father thinks I should do more, but he does not understand how exhausting it is	Intergenerational expectations can lead to misunderstandings and increased stress
Decision-making in healthcare	Primary responsibility	I make all the decisions about her health because I am always with her	Healthcare decision-making often falls solely on the primary caregiver, adding stress to their role
Decision-making in healthcare	Consultation challenges	Sometimes, my siblings disagree on when to take our mother to the doctor	Family disagreements can complicate decision-making and delay timely care
Decision-making in healthcare	Role of medical advice	I rely heavily on the doctor's recommendations for her care plan	Medical advice plays a pivotal role in shaping caregivers' decisions
Decision-making in healthcare	Balancing opinions	Everyone in the family has an opinion on what should be done; it gets overwhelming	Balancing differing family opinions can complicate decision-making for caregivers
Experiences with healthcare	Accessibility issues	Healthcare is hard to access, and appointments are usually rushed	The lack of accessible, patient-centered care adds to the stress faced by caregivers
Experiences with healthcare	Healthcare quality	Doctors often do not take enough time to understand our concerns, and the system feels impersonal	Caregivers frequently feel dismissed by healthcare providers, which undermines trust in the system
Experiences with healthcare	Long wait times	We usually spend hours waiting at the hospital for her checkup	Long waiting times add to the physical and emotional stress of caregiving
Experiences with healthcare	Attitude of medical staff	Sometimes, the nurses are kind, but other times, they are too rushed to listen properly	Inconsistent attitudes of medical staff affect caregivers' trust in healthcare services
Financial responsibility	Hidden costs	It is not just the hospital bills; it is the transportation, medicines, and even the special diet she needs	Financial strain is compounded by hidden costs that are often overlooked in caregiving discussions
Financial responsibility	Personal sacrifices	I have not bought anything for myself in months because all the money goes to her care	Caregivers often forgo personal expenditures to ensure their loved ones receive proper care
Financial responsibility	Budgeting for care	We have had to cut back on other expenses to afford her medications	Caregivers often adjust their household budgets to prioritize healthcare costs
Financial responsibility	Financial dependence	I depend on my husband's income for most of the expenses; it is a lot of pressure on him too	Caregiving can create financial dependency within families, adding strain to relationships
Mental impact and personal sacrifices	Social isolation	I rarely get time for myself or socializing, and it affects my mental health	The isolation experienced by caregivers can have significant mental health implications
Mental impact and personal sacrifices	Burnout	Caregiving is exhausting and mentally draining. I miss the freedom I had before	Burnout is a critical issue for caregivers who face relentless emotional and physical demands
Mental impact and personal sacrifices	Loss of independence	I cannot remember the last time I went out just for fun	The constant demands of caregiving often limit personal freedom and independence
Mental impact and personal sacrifices	Guilt and self-blame	I always wonder if I am doing enough for her, and it keeps me up at night	Feelings of guilt are common among caregivers, even when they are doing their best

The themes and sub-themes presented in Table 2 focus on the complex, layered experience of caregiving. A comparison of the interviews shows that emotional burden, economic deprivation, and structural health barriers were all mentioned by the participants. Despite the difference between caregiving tasks and decision-making, the two themes intersect; those same people who attended to daily activities also held sole responsibility for health-related decisions, which increased stress and emotional tiredness. Results suggest a gendered division of caregiving labor, as male informal care provision is significantly associated with less psychological burden for female caregivers.

There are also thematic intersections that demonstrate how economic limitations lead to psychological suffering. And those who faced higher out-of-pocket costs also reported feeling more burned out and guilty, meaning that economic insecurity is scorchingly direct about making people feel emotionally exposed. For caregivers of loved ones with a deteriorating diagnosis, increasing contact with health professionals, dissatisfaction arising from long waits, and perceived neglect by medical staff were reported more often. These reflections underscore that caregiving hardships in Hyderabad are not isolated but structurally sustained by prevailing gender ideology, financial stress, and insufficient institutional help.

Theme 1: Caregiving Responsibilities

All the participants described caregiving as a responsibility closely related to love and a sense of family unity. They lived by the hour for medication, diet, cleanliness, and assistance in moving. One caregiver said, "My day is all about her and how to take care of what she needs, meals or medicines, or get her around the house."

Many caregivers derived purpose from their role, even in the face of physical and emotional hardship. They say that they derived emotional satisfaction from being able to help, even at personal cost. One participant put it this way: "Even when I am so tired, I just tell myself she needs me. That gives me strength."

Caregivers were frequently sensitive to minute alterations in the care recipient's health status and would identify symptoms long before diagnosis by medical professionals. One said, "I know when something is wrong before my doctor tells me. Even subtle variations in appetite, sleep, or mood tell me when something's off."

Medical crises were another common source of stress. Many who took part talked of having to maneuver their way out of crisis on their own: "She just went down like that, and I had to get her into the hospital by myself in the middle of the night."

Theme 2: The Role of the Other Family Members

This caregiving duty was extremely affected by family dynamics and gender expectations. Most of them were caregiving women who received financial help from less providing male relatives.

Another noted, "My husband provides financially but doesn't help daily." Another said, "My brothers think caregiving is a woman's work. They think I'm going to do everything."

Financial assistance, when provided at all, tended to be intermittent. "My brothers send when they can," said one of the caregivers, "but I am still struggling in meeting all the expenses. Some family support was good." Some siblings were in close touch; others could not be reached: "I have to remind herself and her family that the lockdown is temporary, but she says it's not easy." “My sister gives me encouragement constantly, and that helps keep you going." Another participant remarked, "I wish my family could understand how emotionally tiring this is."

Intergenerational tension developed as younger carers favored modern treatments and older family members were against change. One respondent said, "My own father defies modern treatments, but I wanted him to get the best medical care. It causes tension between us."

Theme 3: Decision-Making in Healthcare

In such cases, caregivers took on the role of exclusive health decision-makers and had to make medical decisions without any previous specialized training or professional direction. This responsibility created considerable stress.

One participant said, "Every single decision is mine; it could be a small issue like allowing the medicine to be changed or a major one like having papa admitted in hospital." Another said, "I have no medical background, and yet, I am called on to decide what is best for her, which is terrifying."

Issues of appointment accessibility, waiting times, and generalism in services were identified frequently. "Appointments are weeks away, and then, when we finally see the doctor, it's over in five minutes," one caregiver said.

Experiences with healthcare providers were frequently not very good. "Others (of us) felt disrespected or disregarded. Doctors expect me to know what to do, but I don't," one said. Another said, "Doctors don't have time for us. I have to ask so many questions for you to get a clear answer."

Caregivers are forced to balance cost versus the quality of treatment. "I want my mother to receive the best care, but private hospitals are expensive, and public hospitals do not have resources we need," said one participant.

Theme 4: Experiences With the Healthcare System

There was a common expression of dissatisfaction with the treatment of the elderly patient and the caregiver in the healthcare system as a whole. The participants often found it challenging to access services due to poor infrastructure, prohibitively high prices, and the shortage of geriatric specialists. A caregiver said, "It takes so much effort just to get her to the hospital, and then, we don't even get proper treatment. The rest of them simply repeated the systemic abuse. It is clear that taking care of the elderly is not a priority in our country." Among other things, the caregivers mentioned the overcrowding of public hospitals and the lengthy engagement booking. One expressed, "We wait hours to see a doctor, and when we finally do, the consultation barely lasts five minutes." Simultaneously, almost all participants mentioned the particularly negative attitude of medical personnel. Some caregivers reported feeling misunderstood or condescended to when they asked questions. One of the participants expressed, "Doctors treat us like we're a nuisance, not real people." Another told, "They don't take what we say seriously, and when we ask questions, they get angry."

Theme 5: Financial Responsibility

Caregiving weighed heavily on their finances. Several caregivers talked of hidden expenditures that grew with time, such as medication, transport, and diet. She said, "I never imagined we'd spend so much on transport alone; it increases so fast." Another person said, "We purchased a wheelchair, built a railing, and ran the heat in her room all the time; all of this harm finances with time." Personal sacrifices are mentioned regularly. A caregiver said, "I haven't bought myself anything in months because every rupee goes on her medicines." Another person said, "I had a dental operation to do, but I postponed it because I can't afford both his operation and my treatment with one another."

To control expenses, families crafted strict schemes of budgeting. "We structure everything around her medical bills; sometimes, we forego things that we need to ensure she has what she needs," one wrote. Others were comparing prices between pharmacies to find ways to save money.

Some caretakers were in a position of financial dependency, causing an emotional burden. One woman said, "I rely on my son for cash, but I feel guilty every time I ask for help." Another wrote, "If my husband decides he can't afford this anymore, I don't know what I'll do."

Theme 6: Psychological Impact and Personal Sacrifices

The psychological effects of caregiving were some of the strongest results. Caregivers felt emotionally drained, anxious, and lonely and had a personal identity crisis.

Social isolation was common. "I can't recall the last time I was able to get together with friends or do something for myself," one participant said. "It's not like it used to be when I could travel out of town at a moment's notice if I wanted to. My world has grown smaller; everything reverts back to caregiving," another added.

Burnout was a recurring theme. "There are days that I feel like giving up, but what else can you do?" one caregiver admitted. "I wake up tired, I sleep tired, and I don't know how long I can keep living like this," said another.

Many had the sense that they were no longer in control of their own destinies. "I had dreams, I had plans, and now, it revolves totally around somebody else's needs," one caregiver said.

Lastly, guilt and self-blame were pervasive in caregiver narratives. "I feel like I'm doing nothing," one confessed.

The results show consistent demographic and thematic associations. Younger caregivers are more resilient, but they struggle with scheduling time because of family or work commitments. Older caregivers, however, reported greater physical burden and social isolation. Among all cases, the emotional exhaustion was consistently the worst burden, in gender, age, and socio-economic contexts. These results highlight the fact that caregiving in resource-poor settings such as Hyderabad does not comprise mere familial obligation but is shaped by larger socio-economic inequities, which ultimately dictate the health of both caregivers and recipients.

## Discussion

This study intended to explore the intricate world of informal caregivers who took care of their elderly dependents with ill health and medications in Hyderabad. The findings demonstrate that what it means to be a caregiver in a low-resource setting is a multifaceted, emotionally and socially restricted position whereby cultural roles and gendered expectations, along with systemic healthcare constraints, all converge. These results provide an important context to the commonplace, everyday lives of caregivers, where it is overwhelmingly mothers and other women who are the main informed carers with little formal support.

In line with other studies, in being at the receiving end of numerous demands and tasks that are "overwhelming," caregivers in this study acknowledged their role as drug managers (of these medications), handlers (to provide mobility assistance), emotional supporters, and medical responders. For most caregivers, the role was all-consuming and even sleep-depriving. This is in line with previous research where current caregiving was associated with fatigue, a lack of social support, and physical fatigue [8,19]. This burden is compounded particularly in Pakistan due to limited formal caregiver training programs and the poor availability of assistive devices [6,20]. Despite this, carers persevered and maintained a high emotional investment, which for many was described as love and duty to support people with dementia at great personal cost in terms of their mental health. Emotional labor has been described as the most frequently experienced source of anxiety, guilt, and burnout among carers [7,10,21,22].

The central role of caregivers in health surveillance and decision-making was identified as a primary theme. Many referred to themselves as "unofficial doctors," having been the first to observe a symptom or change in behavior before even those treating patients. Such vigilance can be constructive, but it is also stressful for the caregivers who themselves are often not prepared for this kind of surveillance and have no medical guidance [23-26]. The absence of proper education support compounds this, highlighting the importance of linking caregiver training programs with local health systems.

One of the most challenging issues caregivers struggled to address was medical emergencies. "Respondents often had to address life-threatening situations on their own without being ready or having anyone else on site reporting directly to them other than the dispatcher," she said. These are traumatic and post-traumatic symptom-related experiences [20,26,27]. These findings demonstrate the need for contextualized emergency preparedness interventions for informal caregivers, perhaps, in theory, particularly for resource-poor contexts where formalized emergency response systems may be less developed [24].

Caregiving roles were also influenced by family configuration and cultural norms. While caring was regarded as a collective (family) undertaking, the division of responsibilities was often unequal, with women doing most of the day-to-day physical caregiving and men providing financial backup. These patterns reflect established gender roles that define child care as part of the woman's domain [6,10,11,20,23]. While an occasional male relative would supply funds, for these caregivers, relatives' money was no substitute for emotional or practical assistance [24,26,27]. Emotional isolation was found in female caregivers having feelings of being orphaned by their brothers or having to decide family matters on their own; this is in accordance with findings that low social support leads to an increase in caregiver [19,21,22].

Intergenerational tensions added another wrinkle to the dynamics between obeying modern medical advice and acceding to traditional remedies favored by elders. Comparable opposing pressures have been reported in other South Asian contexts between evidence-based care and cultural health beliefs [20,23,28]. Not only do these fights result in delays of treatment, but also the frustration that caregivers feel is wedged between a child's obedience and clinical truthfulness.

The decision-making process for health was itself acknowledged as burdensome. Caregivers were often the sole decision-makers, with little or no medical training and institutional support. This is supported by the original studies, in which caregiver freedom in under-resourced settings seems to be associated with high stress and uncertainty [8,19,27]. Obstacles were encountered when seeking advice from healthcare workers in the form of long waiting times, interrupted diagnosis, and minimal communication, which have been reported to be similar among various fractured groups within the Ministry of Health in Pakistan [6,20,25]. Without official medical advice, carers are left prone to misinformation and uncertainty [7,22,26].

Also, the kind of healthcare children got was heavily influenced by financial barriers. Caregivers often had to make trade-offs between cost and care quality, opting for cheaper services that provided inferior results. Economic crisis is consistent with reports, all highlighting the impoverishing effects on access to and making healthcare choices [6,20,23,24].

Discontent with the quality of healthcare is a theme for many respondents who describe services as nonexistent or inadequate and not designed to meet the needs of older patients. These complaints about excessive waiting times, poor treatment, and unfriendly interactions with clinical staff are consistent with published complaints related to other parts of the health infrastructure in Pakistan [6,20,22,25]. This global scarcity directly gives rise to the exacerbation of caregivers' burden and a feeling of powerlessness.

A second large burden was related to the financial aspect of caregiving. Caregivers incurred substantial out-of-pocket cost, and many resorted to loans, family support, or personal savings. In addition, the incidental costs in terms of travel and alterations to the home and extra support devices were particularly difficult [6,23,24,27]. This is in line with previous studies where even monetary sacrifices were expensive from the caregivers' own health and well-being point of view [7,19,22]. Government-funded caregiver support programs or financial cushions are lacking, and these strains compound, giving rise to long-term chronic economic vulnerability, particularly among women [6,11,25,26].

The psychic load on the caregiver was enormous. Social isolation, fatigue, and neglect of self were strongly associated with the anxiety, depression, and chronic stress reported by many caregivers. These findings are in agreement with previous research that has emphasized the impact on mental health of caregiving without institutional or social support [7,19,22]. Guilt and self-blame were also frequent, shaped by perfectionist cultural norms for caregiving [21,22,26]. Over time, this emotional and relational burden led to a loss of the carers' autonomy and sense of themselves as unique individuals who must foreclose their own hopes and dreams, as has been widely observed among caregivers from traditional caregiving cultures [6,11,23,25].

On the whole, these findings highlight that in Pakistan, informal caregiving is situated at the intersection of cultural obligation and structural abandonment. The burden of physical, emotional, and financial hardship on caregivers will remain disproportionate if there are no specific policy efforts, such as caregiver training, financial support programs, or improvement in health service providers' communication skills. Future public health efforts should be directed to building culturally appropriate community-based support systems that enable caregivers and enhance the quality of care for older adults.

This study has certain limitations. It had a small sample size and was performed only in one urban area (Hyderabad), which may restrict the generalization of results to rural areas in Pakistan. Replies were based on self-reported experiences that could be subject to recall or social desirability bias. Furthermore, as this is a qualitative study, it does not measure or assess the quantitative care burden or cause-and-effect relationships. Further mixed-method or longitudinal studies are advised to confirm these findings among a larger sample of people.

## Conclusions

This study aimed to investigate the multidimensional impact of informal caregiving in a primary healthcare setting in Hyderabad, Pakistan, where women assume responsibility for the home-based care of an elderly and/or chronically ill family member. Support through religious and cultural themes to the caregivers who are largely coming from a family setting causes being emotionally drained and economically deprived. These problems are worsened by the lack of institutional support, overpopulated healthcare facilities, and the absence of eldercare services. The results underscore the pressing need for policy makers to acknowledge the importance of caregivers as essential members of healthcare. Community-based respite programs, financial support, and mental health care can help to decrease burnout and improve the well-being of caregivers. Incorporating occupational therapy (OT) and physical therapy (PT) services in geriatric care, combined with training programs and education campaigns, will encourage caregiver support systems. We should further broaden these studies to other demographic and geographic groups in order to gain a deeper understanding of the dynamics around caregiving across Pakistan.
